# Psychosocial correlates of venous thromboembolism-preventive practices among pregnant and postpartum women in China: a cross-sectional structural equation modeling study

**DOI:** 10.3389/fpubh.2026.1903858

**Published:** 2026-08-12

**Authors:** Sixu Liu, Dinara Ospanova, Xiujing Guo, Shenglin Hu, Zhongli Shi

**Affiliations:** 1 Faculty of Medicine and Healthcare, Farabi University, Almaty, Kazakhstan; 2 YAAN People’s Hospital, Yaan, China; 3 Department of Obstetric Nursing, West China Second University Hospital, Sichuan University, Chengdu, China; 4 Key Laboratory of Birth Defects and Related Diseases of Women and Children (Sichuan University), Ministry of Education, Chengdu, China

**Keywords:** health knowledge, attitudes, practice, pregnancy, self-efficacy, structural equation modelling, venous thromboembolism

## Abstract

**Background:**

Venous thromboembolism (VTE) is a major preventable cause of maternal morbidity. Preventive practices may be related to knowledge, attitudes, perceived support, family function, and confidence in performing recommended behaviors. This study examined the psychosocial correlates and prespecified indirect statistical pathways associated with VTE-preventive practices among pregnant and postpartum women in China.

**Methods:**

This hospital-based cross-sectional study was conducted from January to October 2023 among 1,384 pregnant and postpartum women at a tertiary women’s and children’s hospital in Chengdu, China. Participants completed questionnaires assessing venous thromboembolism knowledge, attitudes, preventive practices, self-efficacy, family function, and social support. A latent-variable structural equation modeling, guided primarily by the Information–Motivation–Behavioral Skills (IMB) model, was estimated using 5,000 bootstrap resamples. Multigroup and covariate-adjusted sensitivity analyses were conducted to assess robustness.

**Results:**

Attitude was the strongest correlate of preventive practices (*β* = 0.522, 95% CI 0.463 ~ 0.574). Knowledge (*β* = 0.236, 95% CI 0.166 ~ 0.303), family function (*β* = 0.162, 95% CI 0.091 ~ 0.232), and social support (*β* = 0.185, 95% CI 0.105 ~ 0.258) were positively associated with attitude. The standardized indirect association of knowledge with practice through attitude was *β* = 0.123; the corresponding unstandardized estimate was *B* = 0.481. Self-efficacy had a small association with practice (*β* = 0.065). The model explained 16.4% of the variance in attitude, 3.9% in self-efficacy, and 29.0% in practice. Equality constraints on the structural paths did not worsen model fit between pregnant and postpartum participants (*p* = 0.195), and covariate-adjusted estimates were similar.

**Conclusion:**

Attitude was the strongest psychosocial correlate of self-reported VTE-preventive practices in this sample. Knowledge, family function, and social support showed smaller indirect statistical associations through attitude. These cross-sectional findings do not establish temporal or causal mediation, but they support prospective evaluation of culturally sensitive, attitude-focused educational interventions that also involve family and social support.

## Introduction

Venous thromboembolism (VTE), encompassing deep venous thrombosis and pulmonary embolism, remains a leading preventable cause of maternal morbidity and mortality worldwide (1). Globally, VTE affects approximately 1 ~ 2 cases per 1,000 pregnancies, and the elevated risk persists after delivery (2). In China, a recent meta-analysis of more than 3.8 million pregnancies estimated an incidence rate of 0.13% (3), while national hospital surveillance reported an incidence of 68.2 cases per 100,000 live births (4). National guidance recommends risk assessment and appropriate prophylaxis; among women for whom basic behavioral measures are suitable, hydration, mobilization, and lower limb movement are commonly encouraged (5). However, adherence to preventive recommendations remains incomplete (6).

Preventive practice may be associated with multiple psychosocial and contextual factors. Knowledge provides information about VTE risk and prevention, but information alone may not translate into action (7). Attitudes reflect perceived benefits, safety, and personal relevance; self-efficacy reflects confidence in performing preventive behaviors (8). Family function and perceived social support may reinforce or discourage recommended practices (9). A previous analysis of this cohort also identified gestational stage, education, and prior or proactive VTE learning as correlates of knowledge, attitude, and practice scores (10). In the unique cultural context of China, these influences operate within cultural beliefs such as Bao Tai (11) and postpartum confinement, referred to as Zuo Yue Zi (12), which may favor prolonged rest, as well as family decision-making structures that can shape daily activity (13, 14).

The Information–Motivation–Behavioral Skills (IMB) model was therefore used as the primary theoretical framework (15). In this application, VTE-related knowledge represented information, while attitude, family function, and perceived social support represented individual and interpersonal motivational resources (16). Self-efficacy represented behavioral skills, and self-reported preventive practice was the behavioral outcome. We examined the prespecified associational pathways and interpreted indirect effects as statistical decompositions rather than as evidence of temporal or causal mediation.

## Methods

### Study design and setting

This cross-sectional study was conducted from January to October 2023 at a tertiary women’s and children’s hospital in Chengdu, China.

### Conceptual model specification

**Figure 1:**
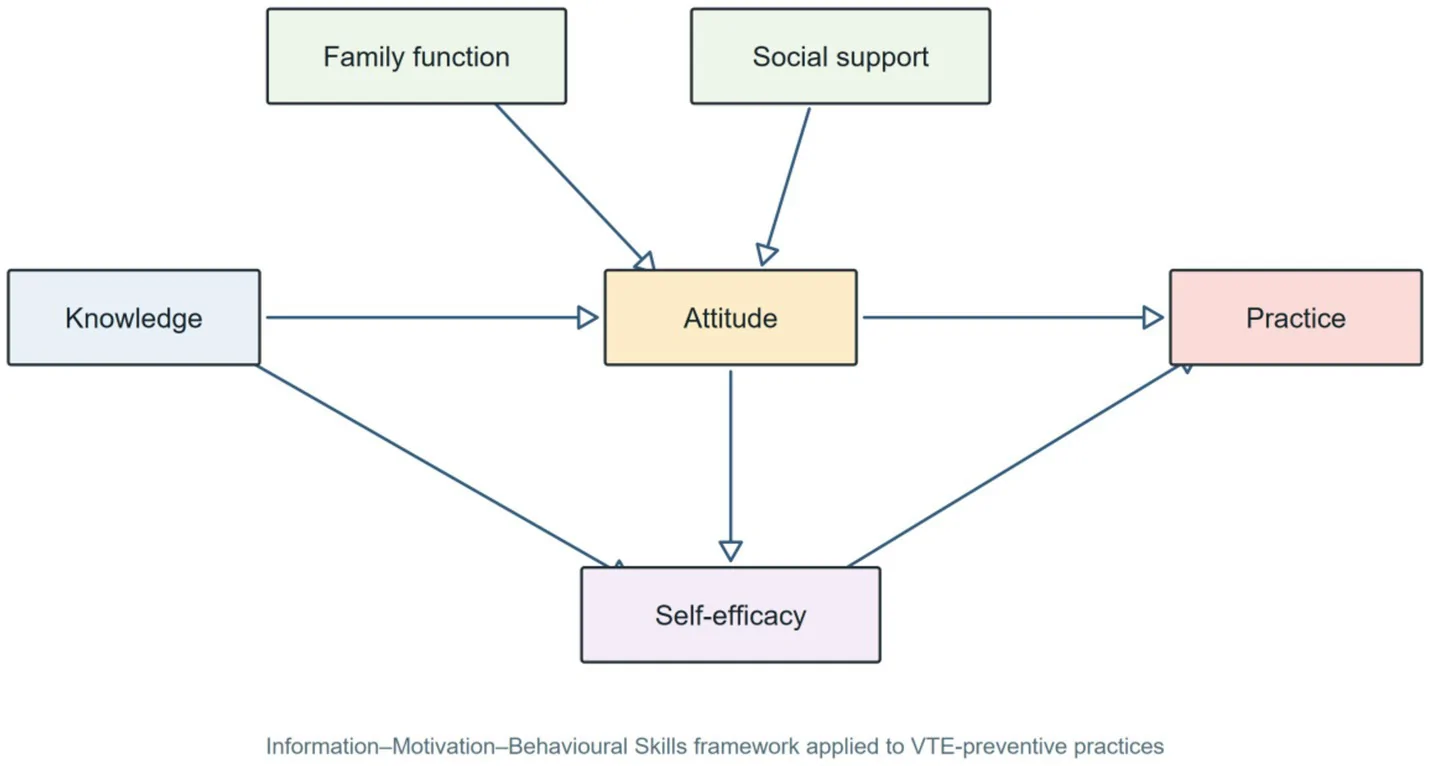
Prespecified conceptual model for VTE-preventive practices.

The tested latent-variable model specified knowledge, family function, and perceived social support as correlates of attitude; knowledge and attitude as correlates of self-efficacy; and attitude and self-efficacy as correlates of preventive practice. Direct knowledge → practice and family function/social support → practice paths were constrained to zero *a priori*. Sociodemographic and obstetric variables were not included in the primary parsimonious structural equation modeling (SEM) because their associations with the Knowledge, Attitudes, and Practices (KAP) domains had been examined in a previously published multivariable analysis of this cohort (10). Figure 1 presents the structure tested in the primary SEM.

### Participants

Eligible participants were pregnant women from the first trimester (≤13 + 6 weeks) through the third trimester (≥28 weeks) and women up to 42 days postpartum who attended antenatal clinics or were hospitalized for obstetric care. Convenience sampling was used. The inclusion criteria were age ≥18 years, no previous VTE diagnosis, and the ability to complete a Chinese questionnaire independently. The exclusion criteria were documented psychiatric or severe cognitive disorders and severe systemic disease, including advanced cardiac, renal, or hepatic dysfunction. Of the 1,415 eligible women invited, 1,384 returned completed questionnaires (participation rate, 97.8%). The characteristics of the 31 non-participants were not collected, so a formal nonresponse comparison was not possible. The sample size calculation for the original survey was reported in the previously published analysis of this cohort (10). Briefly, the formula for estimating a proportion was $n={\left(\frac{{u^{2}}_{\alpha /2}\Pi (1-\Pi )}{\delta^{2}}\right)}^{2}$ . Based on the results from the pilot survey, the pass rates (defined as ≥60% of the total score) for the knowledge, attitude, practice, and total KAP questionnaires were 85, 90, 75, and 86%, respectively. With a margin of error (*δ*) of 0.025 and *α* = 0.05, the maximum estimated sample size was 1,153; inflating this value by 20% yielded a target sample size of 1,384. The achieved sample also exceeded common participant-to-parameter recommendations for the planned SEM.

All questionnaires were completed on paper at the study site with researcher guidance and immediate checking for completeness and consistency. None of the 1,384 returned questionnaires had more than 20% missing responses; therefore, no questionnaire was excluded, and there was no item-level missingness. The cleaned database contained 1,384 complete cases, and no imputation or full-information maximum likelihood procedures were required.

### Measures

#### Sociodemographic and obstetric variables

Recorded sociodemographic variables included maternal age, education, residence, employment, and monthly household income per capita. Education was categorized into six levels, ranging from primary school or below to postgraduate degree. Obstetric variables included pregnancy/postpartum stage, gravidity, parity, varicose veins, *in vitro* fertilization, multiple pregnancy, history of miscarriage or pregnancy termination, and hospitalization during the current pregnancy. Mode of delivery, exact postpartum day, thrombophilia status, and detailed comorbidity data were not available for this analysis.

#### Prior VTE learning

Participants reported whether they had previously received information about VTE, whether they actively sought VTE information, and whether their spouse had done so; each item used a yes/no response with optional categories for information sources.

#### Knowledge, Attitudes, and Practices regarding VTE prevention

The VTE KAP instrument was developed using a two-round Delphi process and psychometrically evaluated, as reported previously (10). The knowledge domain contains 20 items scored as 0 (incorrect), 1 (uncertain), or 2 (correct), yielding a possible score of 0–40. The attitude domain contains 12 items scored from 1 (strongly disagree) to 5 (strongly agree), yielding a possible score of 12–60. The practice domain contains eight items scored from 1 (never) to 5 (always), yielding a possible score of 8–40. Higher scores indicate greater knowledge, more favourable attitudes, and more frequent self-reported preventive practices. No item was reverse-scored.

Prior pilot evaluation among 138 participants from this cohort yielded Cronbach’s alpha values of 0.889, 0.878, and 0.846 for knowledge, attitude, and practice, respectively, with two-week intraclass correlation coefficients ranging from 0.834 to 0.891 (7). These values describe the earlier instrument evaluation. In the full analytical sample used for the present SEM, Cronbach’s alpha values were 0.857 for knowledge, 0.809 for attitude, and 0.918 for practice.

The content domains covered VTE risk factors, symptoms, preventive strategies, perceived severity and benefits, willingness to act, mobility, hydration, and lower limb movement. Measurement model reliability and validity estimates for the full analytical sample are reported in Table 1.

**Table 1:** Measurement model reliability and convergent validity.

Construct	Indicators	Standardized loading range	Cronbach’s alpha	CR	AVE	Sqrt (AVE)
Knowledge	5 parcels (20 items)	0.593–0.851	0.857	0.863	0.562	0.750
Attitude	4 parcels (12 items)	0.762–0.822	0.809	0.870	0.626	0.791
Practice	4 parcels (8 items)	0.787–0.959	0.918	0.930	0.770	0.877
Family function	5 individual items	0.616–0.829	0.879	0.876	0.589	0.767
Self-efficacy	5 parcels (10 items)	0.770–0.878	0.913	0.924	0.708	0.841
Social support	4 parcels (12 items)	0.878–0.964	0.957	0.958	0.850	0.922

CR, composite reliability; AVE, average variance extracted. The Fornell–Larcker criterion was met for all constructs.

#### Self-efficacy

Self-efficacy was measured using the General Self-Efficacy Scale (GSES) developed by Schwarzer et al. (17). The scale comprises 10 items rated on a 4-point Likert scale (1 = not at all true to 4 = exactly true), assessing individuals’ confidence in managing daily challenges. The total score is divided by 10 to yield the mean self-efficacy score, with higher scores indicating greater perceived capability. The Chinese version of the GSES has acceptable psychometric performance (18). Cronbach’s alpha for the present sample was 0.913.

#### Family function

Family function was assessed using the Family APGAR Index developed by Smilkstein (19) and adapted into a Chinese version by Lv et al. (20). The five items evaluate adaptability, partnership, growth, affection, and resolve, with each item scored on a 3-point scale (0 = hardly ever, 1 = some of the time, and 2 = almost always). The instrument has been used in Chinese pregnant populations (21). Cronbach’s alpha for the present sample was 0.879; the measurement model yielded a composite reliability (CR) of 0.876 and an average variance extracted (AVE) of 0.589.

#### Social support

Perceived social support was assessed using the 12-item Multidimensional Scale of Perceived Social Support (MSPSS), referred to as the Perceived Social Support Scale (PSSS) in its Chinese adaptation (22, 23). The instrument consists of 12 items across three dimensions: Family support, friend support, and support from significant others (e.g., colleagues or supervisors). Each item is rated on a 7-point Likert scale ranging from 1 (very strongly disagree) to 7 (very strongly agree). The MSPSS has also shown good reliability and a three-factor structure in pregnant populations (24). Cronbach’s alpha for the present sample was 0.957; composite reliability was 0.958, and average variance extracted was 0.850.

#### Management of data quality

To ensure data quality, research staff received standardized training, and pilot testing was conducted to confirm questionnaire clarity. Completed questionnaires were checked on-site for missing or inconsistent responses, and those with more than 20% missing items were excluded. All data were double-entered using EpiData with logical and range checks, and discrepancies were resolved through verification with the original records.

### Statistical analysis

All statistical analyses were performed using SPSS version 26.0 and AMOS version 29.0. Descriptive statistics were used to summarize sociodemographic and clinical characteristics. Categorical variables were presented as frequencies and percentages, while continuous variables were expressed as means and standard deviations. Statistical significance for all univariate and structural tests was determined using a two-tailed *p*-value of less than 0.05.

The primary SEM was intentionally parsimonious and did not include demographic or clinical covariates. A sensitivity model was used to adjust the attitude, self-efficacy, and practice equations for age, pregnancy/postpartum stage, education, income, gravidity, parity, varicose veins, *in vitro* fertilization, multiple pregnancy, history of miscarriage/pregnancy termination, and hospitalization during the current pregnancy. Age, gravidity, and parity were standardized; pregnancy stage was indicator-coded; education and income were entered as ordered scores; and binary clinical variables were indicator-coded. Pre-pregnancy BMI was excluded because it did not represent contemporaneous body size at the time of the survey across pregnancy and postpartum stages. All covariates were prespecified based on available data; no uncollected clinical variables were imputed.

Maximum likelihood estimation was used for the primary model. As several observed indicators were non-normally distributed, bias-corrected bootstrap confidence intervals based on 5,000 resamples were used for direct and indirect effects. This number of resamples was chosen to stabilize tail quantiles for 95% confidence intervals rather than to increase the apparent precision of point estimates.

Knowledge, attitude, practice, family function, self-efficacy, and social support were modeled as latent variables. The 20 knowledge items were combined into five parcels, the 12 attitude items into four parcels, the eight practice items into four parcels, the 10 GSES items into five parcels, and the 12 MSPSS items into four parcels. The five Family APGAR items were retained as individual indicators. Standardized factor loadings, Cronbach’s alpha, composite reliability (CR), and average variance extracted (AVE) were evaluated. Furthermore, CR > =0.70 and AVE > =0.50 were used as reference values, and discriminant validity was evaluated using the Fornell–Larcker criterion. In total, three correlated residuals (CP3 with CP4, FP3 with FP4, and D1 with D2) were included in the final model and are disclosed because they were added during model refinement. Global model fit was interpreted using the chi-squared test with degrees of freedom, chi-square/df < 5, comparative fit index (CFI) and Tucker–Lewis index (TLI) > =0.90, root mean square error of approximation (RMSEA) < =0.08 with its 90% CI, and SRMR<=0.08 as reference values.

The structural model estimated the seven paths shown in Figure 1. Indirect effects were reported in both unstandardized (B) and standardized (*β*) metrics. *R*^2^ was reported for attitude, self-efficacy, and practice. Harman’s single-factor test was used as a limited diagnostic assessment of common-method variance. Pregnant participants were compared with postpartum participants using multigroup SEM: Configural, metric, and scalar models were evaluated before equality constraints were placed on the seven structural paths.

## Results

Harman’s single-factor test showed that the first unrotated factor accounted for 27.56% of the total variance. This result was used only as a limited diagnostic assessment and does not exclude the possibility of common method bias. The analysis included 1,384 participants (mean age 30.95 ± 3.54 years). Gestational trimesters were evenly distributed (25.0, 25.0, and 28.5%), with 21.5% of participants in the postpartum period (Table 2). Most participants were Han (97.9%), and 56.3% held an undergraduate degree. Key characteristics included low awareness of venous thromboembolism (VTE) (18.2%) and low rates of assisted reproduction (7.9%) and hospitalization (7.9%).

**Table 2:** Sociodemographic and obstetric characteristics of participants (*N* = 1,384).

Variables	Frequency (*N* = 1,384)	Percentage (%)	Knowledge		Attitude		Practice	
			*t*-test/ANOVA	*p*	*t*-test/ANOVA	*p*	*t*-test/ANOVA	*p*
Gestational stage			74.46	**<0.001**	4.51	**0.004**	10.95	**<0.001**
First trimester (≤13 + 6)	346	25.0						
Second trimester (14 ~ 27 + 6)	346	25.0						
Third trimester (>28)	394	28.5						
Postpartum	298	21.5						
Ethnicity			1.82	0.142	0.52	0.672	1.34	0.260
Han	1,355	97.9						
Zang	4	0.3						
Yi	3	0.2						
Others	22	1.6						
Education level			7.01	**<0.001**	1.35	0.237	4.31	**0.001**
Primary school and below	2	0.2						
Junior high school	21	1.5						
High school/technical secondary school	49	3.5						
Junior college	257	18.6						
Undergraduate	779	56.3						
Master or above	276	19.9						
Per capita monthly income/yuan			2.24	**0.029**	1.57	0.141	1.50	0.162
<2000	9	0.7						
2000–2,999	12	0.9						
3,000–4,999	95	6.9						
5,000–7,999	273	19.7						
8,000–9,999	207	15						
10,000–15,000	352	25.4						
15,001–20,000	232	16.8						
>20,000	204	14.7						
IVF			−1.00	0.319	−0.29	0.773	−0.58	0.564
Yes	110	7.9						
No	1,274	92.1						
Pregnancy hospitalization			−1.11	0.271	1.46	0.146	1.60	0.113
Yes	110	7.9						
No	1,274	92.1						
Prior VTE learning			8.24	**<0.001**	4.68	**<0.001**	6.51	**<0.001**
Yes	252	18.21						
No	1,132	81.79						
Proactive learning			7.01	**<0.001**	5.01	**<0.001**	8.59	**<0.001**
Yes	239	17.27						
No	1,145	82.73						
Knowledge	33.49 (5.19)^a^							
Attitude	46.93 (5.04)^a^							
Practice	32.69 (6.54)^a^							

a Mean(SD). Bold values indicate statistical significance (*p* < 0.05).

Correlation analyses revealed significant positive associations among all knowledge, attitude, and practice scores (*ρ* = 0.17–0.55, *p* < 0.001; Table 3). Family function, self-efficacy, and social support were also positively correlated with KAP scores (*ρ* = 0.09–0.30) (Table 3).

**Table 3:** Descriptive statistics and correlations among demographic and psychosocial variables and VTE knowledge, attitude, and practice scores (*N* = 1,384).

Variable	Mean	SD	Median	Min	Max	Knowledge		Attitude		Practice	
						Spearman’s ρ	*p*	Spearman’s ρ	*p*	Spearman’s ρ	*p*
Age	30.95	3.54	31	19	45	0.02	0.464	−0.04	0.1667	0.00	0.9859
Knowledge	33.49	5.19	35.5	8	40	/	/	0.17	**<0.001**	0.22	**<0.001**
Attitude	46.93	5.04	47	12	60	0.17	**<0.001**	/	/	0.55	**<0.001**
Practice	32.69	6.54	33	8	40	0.22	**<0.001**	0.55	**<0.001**	/	/
Family function	8.80	1.93	10	0	10	0.12	**<0.001**	0.21	**<0.001**	0.30	**<0.001**
Self-efficacy	25.27	5.52	25	10	40	0.09	**0.001**	0.16	**<0.0001**	0.17	**<0.0001**
Social support	65.44	11.18	68	12	84	0.13	**<0.001**	0.30	**<0.001**	0.30	**<0.001**

^a^Bold values indicate statistical significance (*p* < 0.05).

The measurement model showed standardized loadings ranging from 0.593 to 0.964 across the six constructs. Cronbach’s alpha values ranged from 0.809 to 0.957, CR from 0.863 to 0.958, and AVE from 0.562 to 0.850; the Fornell–Larcker criterion was met (Table 1). The final structural model had a chi-squared value of 1,335.03 (df = 311), chi-squared/df of 4.29, CFI of 0.964, TLI of 0.959, and RMSEA of 0.049 (90% CI 0.046–0.051). The SRMR was 0.090, which exceeded the 0.08 reference value and indicates residual misfit despite acceptable fit according to the other reported indices (Table 4).

**Table 4:** Global fit indices for the final structural equation modeling.

Index	Reference value	Result
Chi-squared	–	1,335.03
Degrees of freedom	–	311
Chi-squared/df	<5	4.29
CFI	>=0.90	0.964
TLI	>=0.90	0.959
RMSEA (90% CI)	<=0.08	0.049 (0.046–0.051)
SRMR	<=0.08	0.090

The SRMR exceeded the prespecified reference value; the other reported approximate fit indices met their reference values.

**Figure 2:**
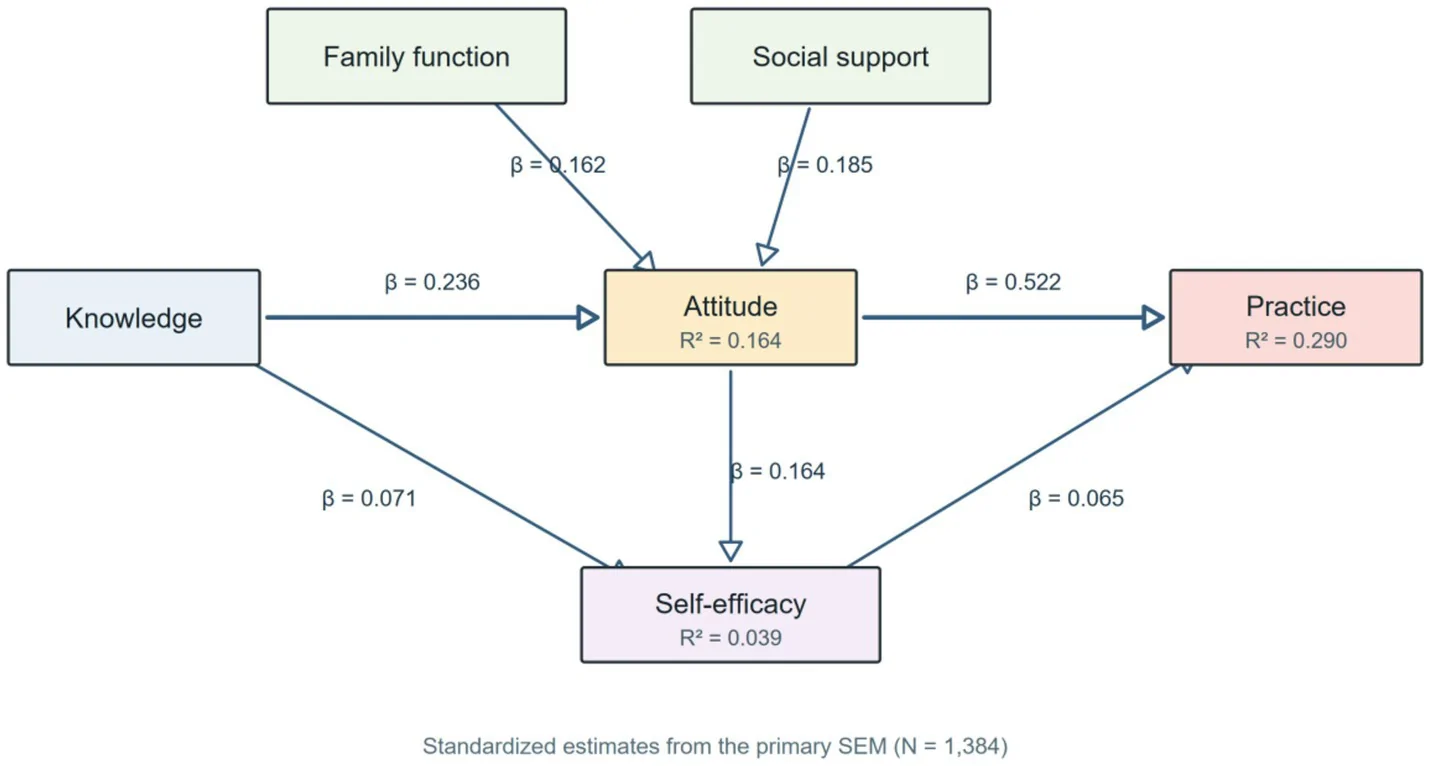
Structural equation modeling with standardized path coefficients.

In the primary model, attitude had the largest standardized association with practice (*β* = 0.522, 95% CI 0.463–0.574, *p* < 0.001). Knowledge (*β* = 0.236, *p* < 0.001), social support (*β* = 0.185, *p* = 0.001), and family function (*β* = 0.162, *p* < 0.001) were associated with attitude. Knowledge (*β* = 0.071, *p* = 0.028) and attitude (*β* = 0.164, *p* < 0.001) were associated with self-efficacy, while the association between self-efficacy and practice was small (*β* = 0.065, *p* = 0.011; Figure 2, Table 5). The model explained 16.4% of the variance in attitude, 3.9% in self-efficacy, and 29.0% in practice.

**Table 5:** Structural path coefficients and bootstrap test results.

Path	Unstandardized estimate (*B*)	Standardized estimate (*β*)	Bias-corrected 95% CI		*p*-value
			Lower	Upper	
Attitude → knowledge	0.394	0.236	0.166	0.303	<0.001
Attitude → social support	0.079	0.185	0.105	0.258	0.001
Attitude →family function	0.220	0.162	0.091	0.232	<0.001
Self-efficacy → knowledge	0.164	0.071	0.007	0.136	0.028
Self-efficacy → attitude	0.225	0.164	0.096	0.229	<0.001
Practice → attitude	1.221	0.522	0.463	0.574	<0.001
Practice → self-efficacy	0.111	0.065	0.015	0.116	0.011

Bootstrap indirect effects are reported in both metrics in Table 6. The knowledge → attitude → practice pathway was *B* = 0.481 (95% CI 0.317–0.652) and *β* = 0.123. The corresponding paths for family function and social support through attitude were *B* = 0.269, *β* = 0.085 and *B* = 0.096, *β* = 0.096, respectively. The sum of the seven defined indirect paths was *B* = 0.881 (95% CI 0.675–1.102) and *β* = 0.315. These quantities represent statistical indirect associations and should not be interpreted as evidence of longitudinal mediation.

**Table 6:** Specific and total indirect effects.

Indirect path	*B*	*β*	Proportion of Total B	Bias-corrected 95% CI		*p*-value
				Lower	Upper	
Knowledge → attitude → practice	0.481	0.123	54.6%	0.317	0.652	<0.001
Knowledge → self-efficacy → practice	0.018	0.005	2.1%	0.002	0.053	0.023
Knowledge → attitude → self-efficacy → practice	0.01	0.003	1.1%	0.003	0.021	0.006
Family function → attitude → practice	0.269	0.085	30.5%	0.147	0.414	<0.001
Family function → attitude→ self-efficacy → practice	0.006	0.002	0.6%	0.002	0.012	0.005
Social support → attitude → practice	0.096	0.096	10.9%	0.054	0.138	0.001
Social support → attitude → self-efficacy → practice	0.002	0.002	0.2%	0.001	0.005	0.006
Total indirect effect	0.881	0.315	100.0%	0.675	1.102	<0.001

*B* denotes an unstandardized effect; beta denotes a standardized effect. Indirect effects are statistical decompositions and do not establish temporal mediation.

Multigroup analysis supported metric invariance between pregnant (*n* = 1,086) and postpartum (*n* = 298) participants (scaled chi-squared difference *p* = 0.380). The scalar comparison was statistically significant (*p* = 0.019), but the change in CFI was −0.0006. Constraining the seven structural paths to equality did not significantly worsen model fit (scaled chi-squared difference = 9.88, df = 7, *p* = 0.195). In the covariate-adjusted sensitivity model excluding BMI, the seven standardized core coefficients were similar to those in the primary model (0.263, 0.183, 0.161, 0.070, 0.163, 0.517, and 0.059 in the same path order); the knowledge → self-efficacy path no longer met the threshold of *p* < 0.05 (*p* = 0.057). Adjusted *R*-squared values were 0.206 for attitude, 0.057 for self-efficacy, and 0.308 for practice. These analyses did not provide strong global evidence that pregnancy/postpartum status or the measured covariates materially altered the central pattern.

## Discussion

In this single-center cross-sectional sample, attitude was the strongest psychosocial correlate of self-reported VTE-preventive practice. Knowledge, family function, and perceived social support showed smaller indirect statistical associations through attitude. Self-efficacy was associated with practice, but its coefficient and explained variance were small. Higher education and active learning experiences were also associated with more favorable KAP profiles. The central role of attitude aligns with evidence from maternal health behavior research, where attitudes commonly emerge as the most proximal predictor of engagement.

Attitude showed the strongest standardized association with preventive behavior. It also acted as the main mediating pathway linking knowledge, family function, and social support to VTE-preventive practices. These findings are consistent with the reasoned action approach, which identifies attitude as a proximal determinant of health actions (25). In the specific context of Chinese maternal populations, this dominant attitudinal pathway may be interpreted in light of the cultural friction between modern medical advice and traditional beliefs such as Bao Tai and Zuo Yue Zi (11, 12). When women perceive that activity restriction is a safer option for fetal or maternal health based on traditional values, their instrumental attitude toward early ambulation becomes negative regardless of their factual knowledge (11). One possible explanation is that venous thromboembolism-preventive behaviors, such as hydration and leg exercises, are low in behavioral complexity. When skill demands are minimal, motivational evaluations typically outweigh competence beliefs (26, 27). This interpretation suggests that clinical interventions should prioritize shifting the attitudinal framework from viewing rest as a safety measure to recognizing activity as an evidence-based preventive strategy. Risk communication that improves women’s understanding of perceived severity and safety can support this process (28).

Although knowledge showed a direct association with related practices (29, 30), its pathway through attitudes appeared more salient within our model. This pattern is consistent with evidence from health behavior research indicating that knowledge alone is insufficient to change behavior (31, 32). This may be because knowledge typically enhances awareness and factual understanding, but these cognitive shifts do not automatically lead to behavioral changes without motivational reinforcement (33). This mechanism aligns with established behavioral theory, which positions attitudes as proximal determinants in the pathway from knowledge to action (34). Attitudes integrate beliefs about severity, benefits, and emotional appraisal, thereby functioning as the interpretive layer that gives meaning to factual knowledge. In the context of VTE, which is an unfamiliar and low-visibility condition for most pregnant women in China and often encounters the cognitive barrier of traditional beliefs, knowledge alone may not be sufficiently compelling (35). Instead, information influences behavior only after it shifts women’s attitudinal evaluations, making the risk feel more personally relevant and the preventive actions more valuable (32). Clinically, these findings suggest that education should integrate knowledge delivery with strategies that strengthen attitudes and risk perception. Visual tools, case-based explanations, and simplified risk communication may help pregnant women better understand the relevance of VTE prevention and engage more actively in protective behaviors.

Demographic and obstetric characteristics were also related to the KAP profiles in the previously published multivariable analysis of this cohort (10). Compared to early pregnancy, knowledge was higher in the second trimester, whereas attitude and practice scores were lower in late pregnancy and the postpartum period. Higher education and proactive VTE learning were associated with better knowledge and/or practices, while maternal age was not retained as an independent correlate. In the present sensitivity analysis, adjustment for available demographic and obstetric variables did not materially change the main SEM associations. These findings suggest that stage-specific education and support may be needed, particularly in late pregnancy and the postpartum period.

Direct paths from family function and perceived social support to practice were not specified in the primary model; both constructs showed indirect statistical associations with practice through attitude. This pattern aligns with prior evidence indicating that supportive interpersonal environments influence health behavior by shaping emotional and cognitive appraisals, including the enhancement of maternal self-efficacy (36, 37). One possible explanation is that family function may enhance feelings of protection and shared responsibility, thereby strengthening positive attitudes toward preventive actions (38). Social support can also buffer stress and reduce psychological barriers that undermine engagement (39). However, in some Chinese households, family members also often act as the primary enforcers of traditional rest-based norms during the perinatal period (12). When family function is characterized by strong adherence to traditional confinement practices, it may inadvertently foster negative attitudes toward active prevention (40). Conversely, supportive interpersonal environments that encourage evidence-based care can buffer cultural stress and reduce psychological barriers (41). These findings highlight the clinical value of involving partners and family members in antenatal education to reinforce positive attitudes and encourage sustained engagement with recommended practices.

Self-efficacy demonstrated a statistically significant but comparatively small effect in our model. It operated mainly as a secondary specified indirect pathway rather than a primary determinant of VTE-preventive behavior. This finding differs from other maternal health behaviors such as gestational diabetes management or physical activity, where self-efficacy often serves as a central predictor (41, 42). A possible explanation is that VTE-preventive actions, such as ambulation, hydration, and adherence to routine clinical advice, involve limited behavioral complexity and require fewer performance-based skills. In low-complexity behaviors, motivational constructs such as attitudes may hold greater influence than confidence in one’s ability to act, a pattern consistent with behavioral theory (27). Self-efficacy may therefore play a reinforcing rather than initiating role in behavioral engagement: Empirical evidence indicates that confidence in one’s ability often rises after individuals have already formed favorable evaluations of an action and perceived its personal relevance (43). This interpretation aligns with the sequential pathways observed in our model, where the effect of knowledge on self-efficacy was largely channeled through attitudes. Clinically, these findings indicate that interventions aimed at strengthening VTE-preventive behavior may benefit from first targeting attitudes and risk perception. Enhancing self-efficacy may still be useful but is likely to be most effective when combined with strategies that build motivation and reinforce the perceived value of preventive actions.

### Strengths and limitations

This study has several strengths. First, it drew on a relatively large sample of pregnant and postpartum women spanning all three trimesters and the postpartum period at a tertiary maternal hospital, which enhances the stability of the estimates and improves the relevance of the findings for clinical settings that manage high-risk pregnancies. Second, the IMB model served as the primary theoretical framework, while the KAP domains and selected TPB concepts were used only for measurement and interpretation. This allowed for a more comprehensive examination of the pathways linking knowledge, attitudes, self-efficacy, and social context to VTE-preventive behavior. Third, the use of structural equation modeling strengthened the analytical rigor by simultaneously estimating multiple direct and indirect effects, while bootstrapping procedures addressed non-normality and provided more robust confidence intervals.

Several limitations should also be considered. The cross-sectional design precludes causal inference, and longitudinal studies are needed to clarify temporal relationships among the constructs. All measures were self-reported, which may introduce social desirability bias and common-method variance. Harman’s single-factor test provided only a limited diagnostic assessment and cannot exclude common method bias. Participants were recruited from a single tertiary hospital, which may limit generalisability to rural settings or primary care populations. Although 1,384 of the 1,415 invited women participated (97.8%), the characteristics of the 31 non-participants were unavailable; therefore, a formal nonresponse comparison could not be conducted. Multigroup analysis did not identify a significant overall difference in structural paths between pregnant and postpartum participants, but the postpartum group was smaller and scalar invariance was not fully supported, so subgroup equivalence should be interpreted cautiously. Mode of delivery, exact postpartum day, thrombophilia, detailed comorbidity data, and contemporaneous BMI were unavailable or not included; therefore, residual confounding cannot be excluded. The Family APGAR, GSES, and PSSS are not pregnancy-specific instruments, although present-sample reliability and measurement model indices were acceptable. Finally, the SRMR was 0.090, indicating some residual model misfit despite acceptable values for the other reported fit indices. In addition, unmeasured factors such as prior medical counselling, cultural beliefs, and partner involvement may have influenced the behavioral pathways but were not captured in this study. Future research should address these limitations and explore the applicability of the model across diverse maternal populations.

## Conclusion

This cross-sectional study found that venous thromboembolism-preventive practices among pregnant and postpartum women were associated with a complex interplay of individual and contextual factors. Attitude emerged as the strongest psychosocial correlate of behavior and represented the principal indirect statistical pathway linking knowledge, family function, and social support with preventive practices. Knowledge showed an indirect statistical association with preventive practice through attitude; a direct knowledge-to-practice path was not estimated in the prespecified model. These findings support prospective evaluation of maternal health education strategies that combine knowledge delivery with attitude-focused and supportive interpersonal approaches. Culturally sensitive risk communication and family involvement may be considered in future interventions, but their effectiveness requires longitudinal or interventional confirmation. Future longitudinal and interventional research is warranted to further refine these behavioral models and to evaluate the effectiveness of culturally adapted programs in strengthening venous thromboembolism prevention within maternal health settings.

## Data Availability

The raw data supporting the conclusions of this article will be made available by the authors, without undue reservation.
